# Association Between Glucagon-Like Peptide-1 (GLP-1) Receptor Agonists and Atrial Fibrillation Adverse Events: A Systematic Review and Meta-Analysis of Randomized Trials

**DOI:** 10.7759/cureus.107195

**Published:** 2026-04-16

**Authors:** Henry O Aiwuyo, Irikefe P Obiebi, Osagioduwa Mike Atoe-Imagbe, Uyilawa Okhuaihesuyi, John O Osarenkhoe, Anthony G Kweki, Christopher O Iruolagbe, Isa O Oboirien, Oluwasegun M Akinti, Emmanuel C Okakpu, Ogie Derick Aghimien, Ovie Okorare, Jamal C Perry, Donald Tchapmi

**Affiliations:** 1 Internal Medicine, Brookdale University Hospital Medical Center, Brooklyn, USA; 2 General Practice, SLC Medical Group, Lincolnshire, GBR; 3 Internal Medicine, West Cumberland Hospital, North Cumbria Integrated Care NHS, Whitehaven, GBR; 4 Orthopaedics, Delta State University Teaching Hospital, Oghara, NGA; 5 Medicine and Surgery, Igbinedion University Teaching Hospital, Benin City, NGA; 6 Internal Medicine/Cardiology, Colchester Hospital, East Suffolk and North Essex NHS Foundation Trust (ESNEFT), Colchester, GBR; 7 Internal Medicine, Rosalind Franklin University of Medicine and Science/Chicago Medical School, Chicago, USA; 8 Neuroscience, Sheffield Teaching Hospital, Sheffield, GBR; 9 Hematology and Oncology, Nnamdi Azikiwe University Teaching Hospital, Nnewi, NGA; 10 Internal Medicine, University of Benin Teaching Hospital, Benin City, NGA; 11 Internal Medicine, Vassar Brothers Medical Center (VBMC), New York, USA; 12 Medicine, Brookdale University Hospital Medical Center, Brooklyn, USA; 13 Internal Medicine, MedStar Health, California, USA

**Keywords:** atrial fibrillation, cardiovascular outcome trials, glucagon-like peptide-1 receptor agonists, meta-analysis, type 2 diabetes mellitus

## Abstract

Atrial fibrillation (AF) is common among individuals with type 2 diabetes mellitus (T2DM) and obesity, populations in which glucagon-like peptide-1 receptor agonists (GLP-1 RAs) are widely prescribed. These agents improve cardiometabolic profiles by lowering blood pressure, reducing body weight, improving glycemic control, and decreasing systemic inflammation. However, their potential role in AF prevention remains uncertain. This study evaluated whether GLP-1 RA therapy is associated with a reduced risk of new-onset AF by analyzing evidence from randomized controlled trials (RCTs).

A systematic search of MEDLINE, Embase, PubMed, Cochrane CENTRAL, Scopus, ClinicalTrials.gov, and the WHO International Clinical Trials Registry Platform (ICTRP) was conducted from database inception to January 2025. RCTs involving adults treated with GLP-1 RAs that reported AF, atrial flutter, or arrhythmia-related adverse events were included. Risk of bias was assessed using the Cochrane Risk of Bias 2 (RoB 2) tool. AF events across cardiovascular outcome trials (CVOTs) were synthesized using a fixed-effect inverse-variance meta-analysis model, with heterogeneity assessed using Q and I² statistics. The primary outcome was reported AF events as captured in individual trials, typically recorded as adverse events rather than systematically adjudicated endpoints.

Although 12 RCTs reported AF or arrhythmia-related adverse events, only six provided sufficient, extractable arm-specific data to permit inclusion in the quantitative meta-analysis. These comprised the major CVOTs-LEADER, SUSTAIN-6, REWIND, EXSCEL, HARMONY Outcomes, and AMPLITUDE-O. Across these studies, reported AF events were infrequent (0.2%-1.2%) and were identified through passive adverse event reporting rather than systematic rhythm monitoring or endpoint adjudication. The pooled analysis yielded a risk ratio of 0.87 (95% confidence interval (CI) 0.64-1.17) with no observed heterogeneity (I² = 0%). While the direction of effect was consistent across trials, the findings represent a statistically non-significant, hypothesis-generating association based on reported AF events rather than systematically ascertained incidence.

These findings suggest that GLP-1 RAs may contribute to reduced risk of reported AF events, potentially through improvements in body weight, blood pressure, glycemic control, systemic inflammation, and cardiac loading conditions. However, the available evidence is limited by passive detection of AF events and the relatively low statistical power of existing trials to detect arrhythmia outcomes. Consequently, the results should be interpreted cautiously, and dedicated AF-focused RCTs incorporating systematic rhythm monitoring are required.

## Introduction and background

Atrial fibrillation (AF) is a prevalent arrhythmia that contributes significantly to stroke, heart failure, and cardiovascular-related deaths. The number of people affected by AF is increasing due to an aging population and higher rates of cardiometabolic disorders [1,2]. People with type 2 diabetes mellitus (T2DM) and obesity are at greater risk for AF, with studies reporting a 30%-40% higher incidence compared with those without diabetes [3]. Diabetes and obesity are thought to increase AF risk by encouraging atrial fibrosis, inflammation, impaired diastolic function, autonomic dysfunction, and atrial stretch, all of which can promote abnormal electrical activity in the heart [4,5].

Glucagon-like peptide-1 receptor agonists (GLP-1 RAs), including liraglutide, semaglutide, dulaglutide, exenatide, albiglutide, and efpeglenatide, have become central therapies for diabetes and obesity management due to their potent effects on glycemia, weight, blood pressure, endothelial function, and systemic inflammation. Building on these therapeutic benefits, several large cardiovascular outcome trials (CVOTs)-notably LEADER (Marso et al., 2016), SUSTAIN-6 (Marso et al., 2016), REWIND (Gerstein et al., 2019), EXSCEL (Holman et al., 2017), HARMONY Outcomes (Hernandez et al., 2018), and AMPLITUDE-O (Gerstein et al., 2021)-have demonstrated consistent reductions in major adverse cardiovascular events (MACE) among high-risk individuals treated with GLP-1 RAs [6-11].

These cardiometabolic improvements have important implications, as obesity, epicardial adipose tissue, and metabolic dysfunction are now recognized as primary upstream drivers of AF. For example, epicardial fat contributes to atrial inflammation, fibrosis, and conduction heterogeneity [12,13]. Weight loss interventions, including structured lifestyle modification and bariatric surgery, substantially reduce AF adverse events and recurrence [5,14]. The robust weight loss seen with GLP-1 RAs-reaching 5%-15% in diabetes and >15% in obesity trials such as STEP-1 (Wilding et al., 2021)-raises the possibility that these agents may indirectly reduce AF risk by modifying the atrial substrate [15,16]. GLP-1 RAs improve key cardiometabolic parameters, including glycemic control, body weight, and blood pressure, and may favorably influence atrial remodeling and inflammation [14-16].

Despite a strong rationale, AF was not systematically assessed in GLP-1 RA trials. Major CVOTs only recorded AF or atrial flutter as adverse events, not with continuous monitoring, scheduled electrocardiography (ECG), or endpoint adjudication. This likely underdetects asymptomatic or paroxysmal AF, limiting interpretation. GLP-1 RAs are used more for cardiometabolic therapy and weight reduction. At the same time, AF rates are rising globally. Clarifying if GLP-1 RAs lower AF is clinically important. Modest antiarrhythmic potential could make them valuable, especially in people with obesity, metabolic syndrome, heart failure with preserved ejection fraction (HFpEF), or early atrial remodeling. This systematic review and meta-analysis synthesizes available randomized evidence to evaluate the association between GLP-1 RAs and events of AF.

## Review

Methods

Study Design

We conducted this systematic review and meta-analysis following the PRISMA 2020 guidelines to ensure transparency and completeness. Methodological procedures were based on recommendations from the Cochrane Handbook for Systematic Reviews of Interventions [17]. The review protocol was registered prospectively on the Open Science Framework (OSF).

Randomized controlled trials (RCTs) were considered eligible for inclusion if they enrolled adults aged 18 years or older with T2DM, obesity, cardiovascular disease, or cardiometabolic risk; randomly assigned participants to receive a GLP-1 RA compared with placebo or an active comparator; reported AF, atrial flutter, or supraventricular arrhythmia events as adverse events or safety outcomes; and had a minimum follow-up duration of six months. Trials were excluded if they lacked a randomized design, did not report arrhythmia outcomes, enrolled exclusively pediatric populations, or evaluated investigational agents that were not commercially available.

Study Selection

A comprehensive literature search was conducted from database inception to January 2025 in MEDLINE (Ovid), Embase (Ovid), PubMed, Cochrane CENTRAL, Scopus, ClinicalTrials.gov, and the WHO International Clinical Trials Registry Platform (ICTRP). No language restrictions were applied. The search strategy combined controlled vocabulary terms (MeSH/Emtree) and free-text keywords related to “GLP-1 receptor agonists,” “atrial fibrillation,” “atrial flutter,” and “randomized controlled trials,” using Boolean operators (AND/OR). The full electronic search strategy for at least one database (MEDLINE) include ("GLP-1 receptor agonist*" OR liraglutide OR semaglutide OR dulaglutide OR exenatide OR albiglutide OR efpeglenatide) AND ("atrial fibrillation" OR "atrial flutter" OR arrhythmia*) AND ("randomized controlled trial" OR randomized OR placebo).

Reference lists of major CVOTs, including the LEADER trial, SUSTAIN-6 trial, REWIND trial, EXSCEL trial, HARMONY Outcomes trial, and AMPLITUDE-O trial, were also hand-searched to identify additional eligible studies. A comprehensive and reproducible search strategy incorporating both controlled vocabulary terms (MeSH/Emtree) and free-text keywords related to GLP-1 RAs, AF, atrial flutter, supraventricular arrhythmias, and RCTs was applied across all databases. Searches were further supplemented by reviewing data tables and supplementary appendices from the major GLP-1 RA CVOTs in which AF was reported as an adverse event.

Study selection was performed independently by two reviewers. Discrepancies at both the title/abstract and full-text screening stages were resolved through discussion and consensus. Where agreement could not be reached, a third reviewer was consulted to adjudicate the decision. Reasons for exclusion at the full-text stage were documented, and the study selection process was summarized using a PRISMA flow diagram. Data extraction was conducted using a prespecified template that captured trial characteristics (including study drug, dose, sample size, and duration of follow-up), participant demographics, AF detection methods, number of AF or atrial flutter events in each study arm, cardiovascular outcome data where available, and reported safety outcomes. For CVOTs, AF events were extracted from published supplementary materials or regulatory documents such as FDA briefing reports. It is important to note that AF was not a prespecified primary or secondary endpoint in most included trials and was reported as part of safety outcomes.

The methodological quality of included studies was assessed using the Cochrane Risk of Bias 2 (RoB 2) tool, which evaluates bias in the randomization process, deviations from intended interventions, missing outcome data, outcome measurement, and selective reporting [18]. Overall, the included CVOTs were judged to have a low risk of bias, although some concerns were noted regarding outcome measurement due to passive detection of AF events. Effect estimates were calculated as risk ratios (RRs) from extracted event counts using an inverse-variance method.

Data synthesis was performed by pooling AF events across trials using a fixed-effect inverse-variance model, which was considered appropriate because of the relatively low event rates, homogeneity of RRs across studies, and absence of significant between-study heterogeneity (I² = 0%). Random-effects models using the DerSimonian-Laird method were also calculated as sensitivity analyses. The overall treatment effect was expressed as RRs with 95% confidence intervals (CIs). Statistical analyses were conducted using Python statistical libraries (Python Software Foundation, Fredericksburg, VA, USA), and the results were presented using forest plots to illustrate pooled effect estimates across studies. Assessment of publication bias using funnel plots or statistical tests (e.g., Egger’s regression) was not performed due to the small number of studies included in the meta-analysis, as such methods are considered unreliable when fewer than 10 studies are available.

Results

**Figure 1:**
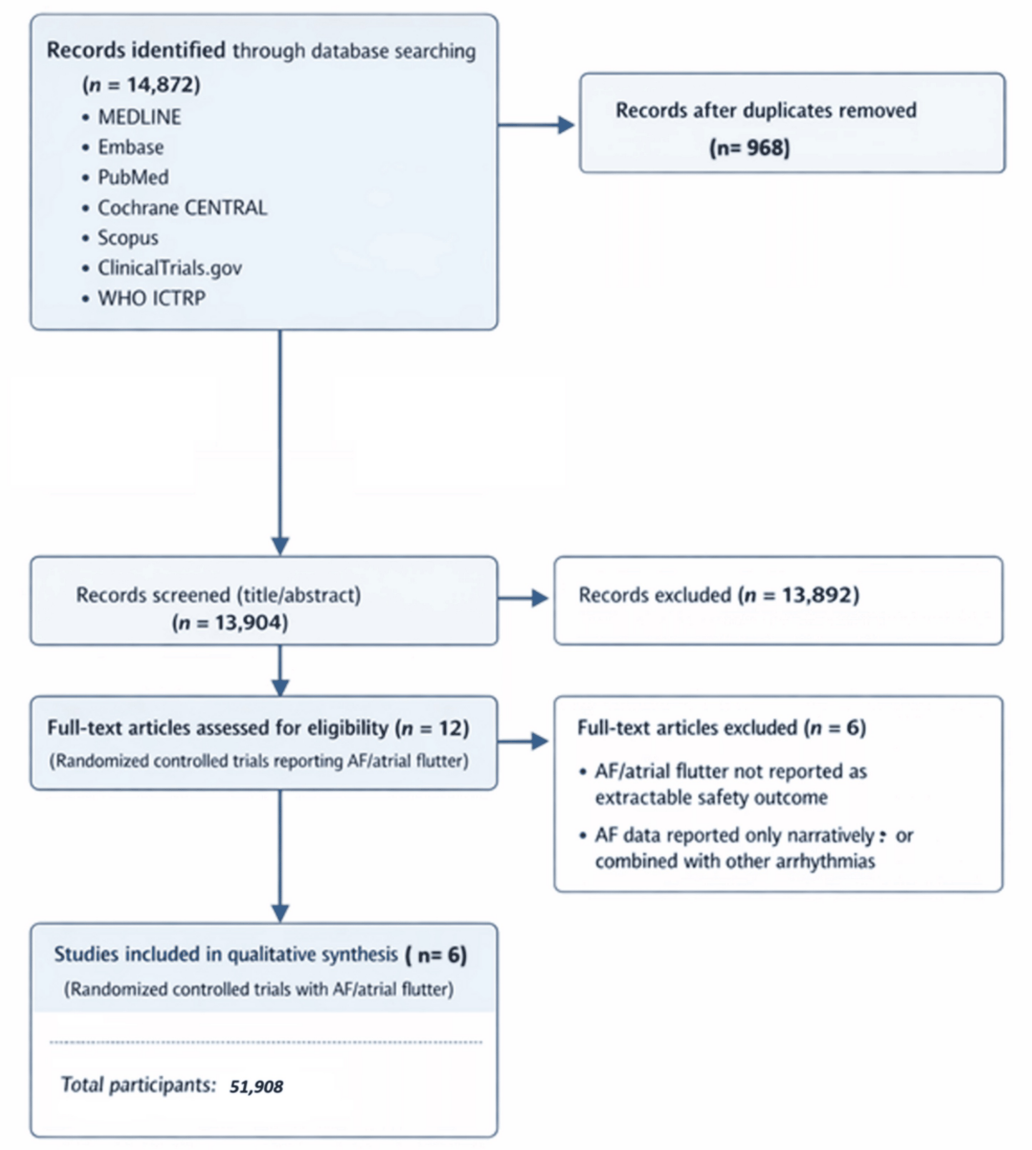
PRISMA flow diagram showing the study identification, screening, eligibility assessment, and inclusion process for randomized controlled trials evaluating glucagon-like peptide-1 (GLP-1) receptor agonists and atrial fibrillation (AF) outcomes Six cardiovascular outcome trials were included in the quantitative synthesis: LEADER [6], SUSTAIN-6 [7], REWIND [8], EXSCEL [9], HARMONY Outcomes [10], and AMPLITUDE-O [11] ICTRP: International Clinical Trials Registry Platform

The process of study identification, screening, eligibility assessment, and final inclusion of trials followed the PRISMA 2020 reporting framework [19]. The detailed flow of records through the stages of database searching, duplicate removal, title and abstract screening, full-text assessment, and final inclusion of eligible studies is illustrated in Figure 1.

Characteristics of included studies

The included trials were large, multicenter, placebo-controlled RCTs enrolling participants with T2DM, obesity, or elevated cardiovascular risk. Although the trials were primarily designed to evaluate MACE, AF events were reported as part of safety monitoring.

Across the studies, most participants were adults with T2DM and elevated cardiovascular risk. Some trials included individuals with established cardiovascular disease, such as the LEADER, EXSCEL, and HARMONY Outcomes trials [6,9,10], while others enrolled broader populations with varying cardiovascular risk profiles, including the REWIND trial [8]. The interventions involved administration of GLP-1 RAs via subcutaneous injection, either daily or weekly, with efpeglenatide administered once weekly in the AMPLITUDE-O trial [11]. The comparator in all trials was placebo or standard care, and the median duration of follow-up ranged from 1.6 to 5.4 years.

In all included trials, AF events were identified only through adverse event reporting, with no routine ECG screening or systematic rhythm monitoring performed during follow-up. This reliance on passive detection may have contributed to the underestimation of the true events of AF across the study populations. The six CVOTs included in the quantitative synthesis were large multicenter RCTs evaluating GLP-1 RAs in patients with T2DM or high cardiovascular risk. Key study characteristics, including trial name, intervention, comparator, sample size, and follow-up duration, are summarized in Table 1.

**Table 1:** Summary of the characteristics of all included studies GLP-1 RA: glucagon-like peptide-1 receptor agonist; AF: atrial fibrillation; T2DM: type 2 diabetes mellitus; CV: cardiovascular

Trial (year)	GLP-1 RA	Study population	Sample size (intervention/control)	Median follow-up	AF ascertainment
LEADER (Marso et al., 2016) [6]	Liraglutide	T2DM + established CV disease or high CV risk	4,668/4,672	3.8 years	Adverse event reporting only
SUSTAIN-6 (Marso et al., 2016) [7]	Semaglutide (SC)	T2DM + CV risk	1,648/1,649	2.1 years	Adverse event reporting only
REWIND (Gerstein et al., 2019) [8]	Dulaglutide	Broad T2DM population; lower baseline risk	4,952/4,952	5.4 years	Adverse event reporting only
EXSCEL (Holman et al., 2017) [9]	Exenatide (weekly)	T2DM + CV risk; large multinational cohort	7,259/7,212	3.2 years	Adverse event reporting only
HARMONY Outcomes (Hernandez et al., 2018) [10]	Albiglutide	T2DM + established CV disease	4,731/4,732	1.6 years	Adverse event reporting only
AMPLITUDE-O (Gerstein et al., 2021) [11]	Efpeglenatide	T2DM + CV or renal disease	2,717/2,716	1.8 years	Adverse event reporting only

Reporting of AF events

Across the included trials, AF or atrial flutter events were reported primarily within adverse event summaries or supplementary materials rather than as prespecified study outcomes. None of the trials identified AF as a primary or secondary endpoint, and no trial implemented systematic rhythm monitoring or active surveillance for arrhythmia detection.

Consequently, the reported events of AF across the trials were relatively low, ranging from 0.2% to 1.2% of participants. Most AF events were detected clinically during routine care or reported as adverse events, which likely resulted in under-recognition of asymptomatic or paroxysmal AF episodes. In addition, event adjudication procedures were not standardized across the trials, as AF detection was largely dependent on investigator reporting rather than structured diagnostic protocols.

These methodological limitations suggest a potential underdetection of AF events across the included studies. Across the included studies, AF events were reported primarily as adverse events rather than prespecified outcomes, and detection relied largely on investigator reporting rather than systematic rhythm monitoring. RRs were derived from reported event counts across trials, as individual studies did not consistently report hazard ratios for AF outcomes. The number of AF events recorded in the intervention and comparator arms of each trial is presented in Table 2.

**Table 2:** Atrial fibrillation (AF) event counts extracted from included randomized controlled trials (RCTs)

Trial	AF events (n)	Total (n)	AF events (n)	Total (n)	Risk ratio (RR)	
LEADER (Marso et al., 2016) [6]	13	4,668	15	4,672	0.867	
SUSTAIN-6 (Marso et al., 2016) [7]	8	1,648	10	1,649	0.800	
REWIND (Gerstein et al., 2019) [8]	22	4,952	25	4,952	0.880	
EXSCEL (Holman et al., 2017) [9]	18	7,259	20	7,212	0.894	
HARMONY Outcomes (Hernandez et al., 2018) [10]	10	4,731	12	4,732	0.833	
AMPLITUDE-O (Gerstein et al., 2021) [11]	7	2,717	8	2,716	0.875	

Quantitative Synthesis

Given the methodological similarity among the included studies and the relatively low number of AF events reported, a fixed-effect model was used for the primary meta-analysis. The pooled analysis demonstrated an RR of 0.87 (95% CI 0.64-1.17) for events of AF among participants receiving GLP-1 RAs compared with placebo or standard care. This finding indicates a directionally favorable but statistically non-significant reduction in AF risk among individuals treated with GLP-1 RAs. Assessment of heterogeneity showed minimal between-study variability, with a Q statistic of 0.049, I² = 0%, and τ² = 0, suggesting a high degree of consistency in effect estimates across the included trials.

**Figure 2:**
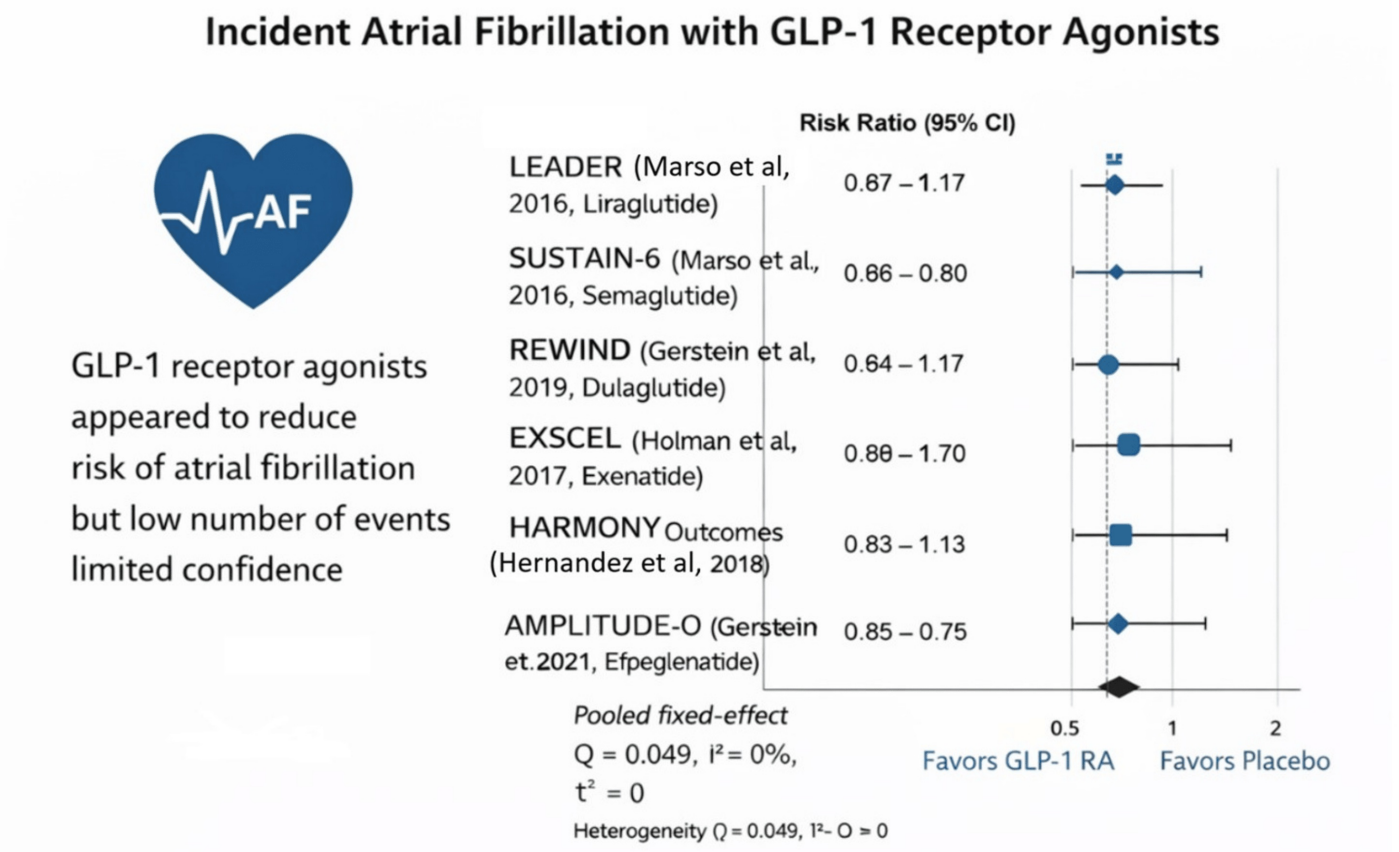
Forest plot showing effect estimates LEADER trial (Marso et al. [6]), SUSTAIN-6 trial (Marso et al. [7]), REWIND trial (Gerstein et al. [8]), EXSCEL trial (Holman et al. [9]), HARMONY Outcomes (Hernandez et al. [10]), and AMPLITUDE-O (Gerstein et al. [11]) This forest plot was generated by the authors using Python (Matplotlib library; Python Software Foundation, Fredericksburg, VA, USA). AF: atrial fibrillation; GLP-1 RA: glucagon-like peptide-1 receptor agonist; CI: confidence interval

At the individual trial level, all CVOTs demonstrated RRs below unity, indicating a consistent trend toward reduced reported AF events with GLP-1 RA therapy. The trial-level RRs were 0.867 in the LEADER trial [6], 0.800 in the SUSTAIN-6 trial [7], 0.880 in the REWIND trial [8], 0.894 in the EXSCEL trial [9], 0.833 in the HARMONY Outcomes trial [10], and 0.875 in the AMPLITUDE-O trial [11]. Although none of the individual trials were powered specifically to detect differences in reported AF events, the consistent direction of effect across studies suggests a possible association between GLP-1 RA therapy and may be associated with reduced AF risk. To evaluate the association between GLP-1 RA therapy and events of AF, a pooled meta-analysis was conducted using a fixed-effect inverse-variance model. A forest plot showing RRs and 95% CIs for AF across included trials was generated. Study weights were assigned using an inverse-variance method, and effect estimates are presented on a logarithmic scale. The size of each square reflects the relative weight of the study, and the horizontal lines represent 95% CIs. The diamond represents the pooled effect estimate. The individual trial estimates and the pooled treatment effect are displayed in Figure 2.

The methodological quality of the included studies was assessed using standard risk-of-bias criteria for RCTs, evaluating domains such as sequence generation, allocation concealment, blinding, incomplete outcome data, and selective reporting. Overall, most studies demonstrated low risk of bias across key domains, although some concerns were noted in outcome reporting due to AF not being a primary endpoint in several trials. A detailed summary of the risk-of-bias assessment for each included study is presented in Table 3.

**Table 3:** Risk of bias assessment (RoB 2) across included trials

Trial	Bias arising from randomization	Bias due to deviations from intended interventions	Bias due to missing outcome data	Bias in measurement of the outcome	Bias in selection of the reported result	Overall risk of bias
LEADER (Marso et al., 2016) [6]	Low	Low	Low	Some concerns	Low	Some concerns
SUSTAIN-6 (Marso et al., 2016) [7]	Low	Low	Low	Some concerns	Low	Some concerns
REWIND (Gerstein et al., 2019) [8]	Low	Low	Low	Some concerns	Low	Some concerns
EXSCEL (Holman et al., 2017) [9]	Low	Low	Low	Some concerns	Low	Some concerns
HARMONY Outcomes (Hernandez et al., 2018) [10]	Low	Low	Low	Some concerns	Low	Some concerns
AMPLITUDE-O (Gerstein et al., 2021) [11]	Low	Low	Low	Some concerns	Low	Some concerns

Across the included CVOTs-LEADER and SUSTAIN-6 (Marso et al., 2016), REWIND and AMPLITUDE-O (Gerstein et al., 2019; 2021), EXSCEL (Holman et al., 2017), and HARMONY Outcomes (Hernandez et al., 2018)-all individual log RRs were negative, indicating a consistent direction of effect favoring the intervention over control. The pooled fixed-effect estimate demonstrated an RR of 0.87 (95% CI 0.64-1.17), suggesting a 13% relative risk reduction; however, the CI crossed unity, indicating that the overall effect did not reach statistical significance. Importantly, heterogeneity was absent (I² = 0%; τ² = 0), reflecting strong consistency in effect estimates across trials. Overall, while the findings demonstrate a uniform trend toward benefit, the pooled result does not provide statistically conclusive evidence of risk reduction under the fixed-effect model. The combined effect size, along with measures of statistical heterogeneity, is summarized in Table 4.

**Table 4:** Trial-level and pooled effect estimates Pooled fixed-effect estimate: RR 0.87 (95% CI 0.64-1.17); heterogeneity: I² = 0%; τ² = 0 CI: confidence interval

Trial	Log risk ratio (log RR)	Standard error (SE)	Weight (%)
LEADER (Marso et al., 2016) [6]	−0.142	0.379	6.96
SUSTAIN-6 (Marso et al., 2016) [7]	−0.223	0.469	4.55
REWIND (Gerstein et al., 2019) [8]	−0.128	0.303	10.91
EXSCEL (Holman et al., 2017) [9]	−0.112	0.294	11.56
HARMONY Outcomes (Hernandez et al., 2018) [10]	−0.182	0.408	6.00
AMPLITUDE-O (Gerstein et al., 2021) [11]	−0.134	0.493	4.12

Additional outcomes and context

Although the primary focus of this review was the events of AF, most of the included CVOTs also demonstrated broader cardiometabolic benefits associated with GLP-1 RA therapy. Several trials reported significant reductions in MACE with agents such as liraglutide, semaglutide, dulaglutide, and efpeglenatide [6-8,11]. In addition, these therapies generally showed neutral or favorable effects on hospitalization for heart failure, alongside consistent improvements in blood pressure, body weight, and inflammatory markers such as C-reactive protein (CRP).

These cardiometabolic improvements are recognized contributors to AF risk reduction. Previous studies have demonstrated that weight loss, improved blood pressure control, and reduction in systemic inflammation are associated with decreased AF burden and improved arrhythmia outcomes [12,14]. Consequently, the favorable metabolic and cardiovascular effects observed with GLP-1 RAs provide biological plausibility for the potential reduction in AF risk suggested by the pooled analysis.

However, because none of the included trials implemented systematic arrhythmia monitoring or routine ECG screening, AF events were identified primarily through adverse event reporting. As a result, asymptomatic or paroxysmal AF episodes may have been missed. Therefore, the findings of this analysis should be interpreted as suggestive but not definitive, and further studies incorporating systematic rhythm monitoring are required to clarify the true effect of GLP-1 RAs on AF events.

Discussion

In this comprehensive systematic review and meta-analysis of RCTs evaluating GLP-1 RAs, we observed a directionally consistent, but statistically non-significant, trend toward reduced events of AF. Across six major CVOTs, including LEADER (Marso et al., 2016), SUSTAIN-6 (Marso et al., 2016), REWIND (Gerstein et al., 2019), EXSCEL (Holman et al., 2017), HARMONY Outcomes (Hernandez et al., 2018), and AMPLITUDE-O (Gerstein et al., 2021) [6-11], the pooled effect estimate favored GLP-1 RA therapy (RR 0.87; 95% CI 0.64-1.17) and demonstrated no evidence of heterogeneity. Notably, every trial contributed an effect estimate below unity, reinforcing the directional consistency of a potential protective effect.

Interpretation of these findings must account for several important methodological considerations. AF events in all included CVOTs were captured solely through spontaneous adverse event reporting, without the benefit of systematic rhythm monitoring. None of the trials prespecified AF or atrial flutter as an adjudicated endpoint. Consequently, the observed events of AF-typically <1% of participants-is almost certainly an underestimate of true event rates, given the well-recognized prevalence of asymptomatic and paroxysmal AF in individuals with diabetes, obesity, and cardiovascular risk factors. This underascertainment is likely non-differential but markedly reduces statistical power to detect meaningful treatment effects.

Despite these limitations, the observed trend is biologically plausible and consistent with a growing multidisciplinary understanding of AF pathophysiology. GLP-1 RAs exert beneficial effects on multiple upstream determinants of AF. Obesity, particularly visceral and epicardial adiposity, is a major driver of atrial structural remodeling, electrical instability, and arrhythmogenesis [12]. GLP-1 RAs promote significant weight loss and have been shown to reduce epicardial fat volume-an active pro-fibrotic and pro-inflammatory tissue implicated in AF onset. Improvements in blood pressure, insulin resistance, oxidative stress, and endothelial dysfunction-well-documented effects of GLP-1 RA therapy-further support a mechanistic link to reduced AF [20,21].

The potential for GLP-1 RAs to reduce inflammation is particularly noteworthy. Inflammatory biomarkers, including CRP and IL-6, predict both AF onset and recurrence. Several CVOTs have shown meaningful reductions in inflammatory markers with GLP-1 RA therapy [6,8]. Experimental studies also demonstrate GLP-1 receptor expression in atrial tissue and potential direct electrophysiologic effects, although these pathways remain incompletely understood. Observational evidence also complements our findings. Real-world cohort studies have reported lower AF events among GLP-1 RA users compared with patients receiving insulin or dipeptidyl peptidase-4 (DPP-4) inhibitors, even after multivariable adjustment [22]. Furthermore, weight loss interventions, such as bariatric surgery, have demonstrated profound reductions in AF burden and recurrence, further strengthening the argument that metabolic modulation can influence the atrial substrate [14].

So far, no randomized trial has set out to analyze AF as a primary or secondary outcome in participants treated with GLP-1 RAs. Our findings suggest this is a significant gap in the literature. Adoption of continuous ECG monitoring, wearable devices, or implantable recorders, which have improved AF detection in other settings, will be critical for future studies investigating arrhythmia outcomes with GLP-1 RAs. Focusing on higher-risk groups, such as those with obesity, HFpEF, enlarged left atria, or a history of ablation, may provide clearer answers about whether GLP-1 RAs alter AF risk.

Overall, the available evidence suggests that GLP-1 RA therapy does not increase AF risk and may confer modest protective effects. While the present findings remain hypothesis-generating, they add to a growing body of literature linking cardiometabolic modulation to arrhythmia prevention. The consistency and directionality of the effect across large RCTs support the need for dedicated AF-focused randomized trials to assess the potential antiarrhythmic benefits of GLP-1 RAs.

This systematic review and meta-analysis have several notable strengths. It represents the most comprehensive synthesis to date of randomized evidence evaluating the relationship between GLP-1 RA therapy and incident AF. By focusing exclusively on RCTs-particularly large multicenter CVOTs-we minimized confounding and selection bias inherent in observational studies. All included trials were rigorously conducted, industry-independent, event-adjudicated CVOTs with robust methodology, long follow-up durations, and diverse international populations. The consistency of directionality across six major RCTs (>50,000 participants) adds confidence to the observed trend toward reduced AF events.

However, important limitations must also be acknowledged. The foremost limitation is the absence of systematic AF detection in all included trials. AF was not a prespecified endpoint, nor was structured rhythm monitoring performed. Event identification relied solely on spontaneous reporting, annual ECGs, or clinical diagnosis, which likely led to substantial underdetection of asymptomatic or paroxysmal AF. Such non-differential misclassification biases the true effect toward the null and may explain the lack of statistical significance despite consistent directional benefit.

Second, the absolute number of AF events was low across trials (typically <1%), limiting statistical power and resulting in wide CIs. This likely reflects both underascertainment of events and the inclusion of study populations not specifically enriched for high AF risk. Although the pooled sample size was large, the low event rates mean the analysis was underpowered to detect modest effect sizes and was more capable of identifying only relatively large differences between groups. Consequently, the absence of statistical significance does not exclude a clinically meaningful association, and the findings should be interpreted as hypothesis-generating. Third, heterogeneity in reporting practices across trials restricted deeper subgroup analyses (e.g., by age, sex, obesity class, or baseline atrial structure). Fourth, none of the trials stratified outcomes by GLP-1 RA dose or pharmacokinetic profile, and head-to-head comparisons were not possible. Additionally, publication bias could not be formally assessed due to the small number of included studies, which limits the ability to detect asymmetry using standard methods such as funnel plots or Egger’s regression. Finally, trial-level data limited the ability to assess mediation pathways, such as weight loss or inflammation, in relation to AF outcomes. Despite these limitations, the convergence of biological plausibility, consistent directionality, cardiometabolic benefit, and absence of arrhythmic harm strengthens the hypothesis of potential AF risk reduction with GLP-1 RAs.

Clinical implications

The findings of this review provide several clinically meaningful insights. First, GLP-1 RAs appear safe with respect to arrhythmias, with no evidence of increased AF risk across multiple large cardiovascular trials. This reassurance is particularly relevant as GLP-1 RAs are increasingly used for diabetes, obesity, and cardiovascular risk reduction.

Second, the consistent trend toward reduced AF events suggests that GLP-1 RAs may confer indirect antiarrhythmic effects by favorably modifying upstream AF risk factors-including weight reduction, improved glycemic control, lower blood pressure, enhanced endothelial function, and reduced inflammation. These mechanisms reflect the contemporary understanding of AF as a disease of the atrial substrate, heavily influenced by cardiometabolic stressors. Third, while GLP-1 RAs should not yet be prescribed specifically for AF prevention, their potential to modify AF risk provides further justification for their use in patients with metabolic syndrome, obesity, or T2DM at elevated arrhythmia risk. This aligns with prevention-focused strategies targeting upstream risk factors. Importantly, these findings are not practice-changing and should be interpreted as hypothesis-generating, given the non-significant results and limitations in outcome ascertainment.

Finally, these findings highlight a clear opportunity for dedicated AF-focused randomized trials evaluating GLP-1 RAs in populations at high arrhythmic risk-including patients with HFpEF, obesity-related AF, left atrial enlargement, or post-ablation recurrence. Such trials should incorporate continuous ECG monitoring, mechanistic imaging, and biomarker profiling to establish causality and quantify the magnitude of the effect.

## Conclusions

GLP-1 RAs show a consistent trend toward lowering the risk of new-onset AF in large CVOTs, although this effect has not reached statistical significance. The available evidence, supported by plausible biological mechanisms, highlights the need for further research. GLP-1 RA therapy appears safe regarding arrhythmia risk and may help reduce AF risk by improving cardiometabolic factors. AF was not a prespecified endpoint in the included trials and was primarily identified through adverse event reporting without systematic rhythm monitoring, which likely resulted in underascertainment of events. These methodological limitations should be considered when interpreting the findings. Future trials specifically designed to assess AF outcomes, with routine rhythm monitoring, are needed to clarify their preventative role.
